# Recent advances and perspectives on supramolecular radical cages

**DOI:** 10.1039/d1sc01618k

**Published:** 2021-09-02

**Authors:** Bin Huang, Lijun Mao, Xueliang Shi, Hai-Bo Yang

**Affiliations:** Shanghai Key Laboratory of Green Chemistry and Chemical Processes, School of Chemistry and Molecular Engineering, East China Normal University 3663 N. Zhongshan Road Shanghai 200062 P. R. China xlshi@chem.ecnu.edu.cn hbyang@chem.ecnu.edu.cn

## Abstract

Supramolecular radical chemistry has been emerging as a cutting-edge interdisciplinary field of traditional supramolecular chemistry and radical chemistry in recent years. The purpose of such a fundamental research field is to combine traditional supramolecular chemistry and radical chemistry together, and take the benefit of both to eventually create new molecules and materials. Recently, supramolecular radical cages have been becoming one of the most frontier and challenging research focuses in the field of supramolecular chemistry. In this *Perspective*, we give a brief introduction to organic radical chemistry, supramolecular chemistry, and the emerging supramolecular radical chemistry along with their history and application. Subsequently, we turn to the main part of this topic: supramolecular radical cages. The design and synthesis of supramolecular cages consisting of redox-active building blocks and radical centres are summarized. The host–guest interactions between supramolecular (radical) cages and organic radicals are also surveyed. Some interesting properties and applications of supramolecular radical cages such as their unique spin–spin interactions and intriguing confinement effects in radical-mediated/catalyzed reactions are comprehensively discussed and highlighted in the main text. The purpose of this *Perspective* is to help students and researchers understand the development of supramolecular radical cages, and potentially to stimulate innovation and creativity and infuse new energy into the fields of traditional supramolecular chemistry and radical chemistry as well as supramolecular radical chemistry.

## Abstract 2

This *Perspective* summarizes the recent developments of supramolecular radical cages including the design and synthesis of radical cages, their interesting host–guest spin–spin interactions and applications in radical-mediated/catalyzed reactions.

## Graphical abstract

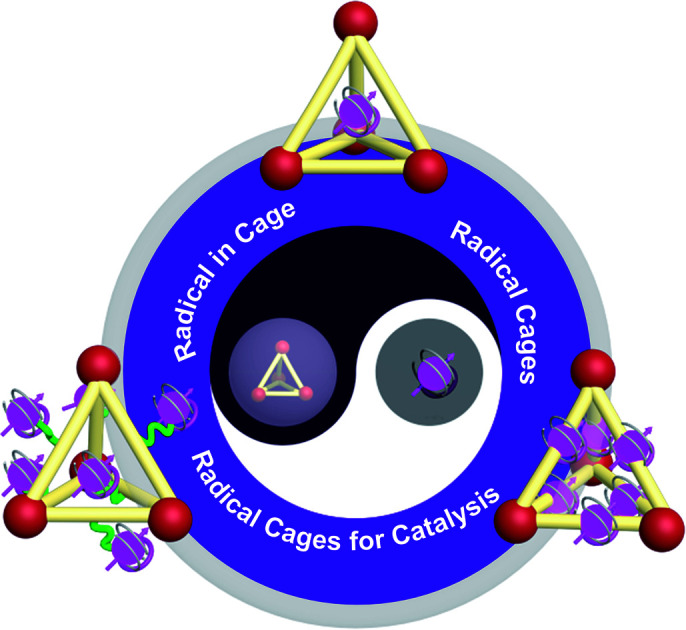

## 1. Introduction

In 1900, Gomberg discovered the first stable organic radical, namely the triphenylmethyl radical, and in that moment a new age opened for the field of organic radical chemistry.^1^ Nowadays, research on organic radicals has been increasingly attracting a great deal of attention and interest from both academia and industry, greatly promoting the development of organic radical chemistry. It is fair to say that the discipline of organic radical chemistry with wide application and scientific implication has penetrated to chemistry, physics, biology, medicine, materials science, and many other disciplines and application areas.^2^ Supramolecular chemistry is a very active research field that has been growing and prospering since its emergence in the last century. Supramolecular chemistry aims at developing highly complex chemical systems and advanced functional materials using supramolecular self-assembly of various components by means of noncovalent interactions which is markedly distinct from conventional covalent chemistry.^3^ Supramolecular chemistry has become one of the most interdisciplinary fields, crossing a range of disciplines from organic chemistry, physical chemistry, polymer chemistry, and coordination chemistry to materials science, nanotechnology and biological science.^4^ Indeed, the importance of supramolecular chemistry has been recognized by the Nobel Prize winning work in 1987 and 2016.^5^

Considering the importance of radical chemistry and supramolecular chemistry and their distinctly interdisciplinary characteristics, it is conceivable that their combination has significant consequences where both fields may furnish synergistic help in the establishment of some new concepts and new research subjects. Supramolecular radical chemistry, naturally, is emerging as a cutting-edge interdisciplinary field of traditional supramolecular chemistry and radical chemistry that has grown considerably in recent years. Supramolecular radical chemistry first appeared as a term in 2012 in one chapter of *Encyclopedia of Radicals in Chemistry, Biology and Materials*, wherein Prof. Marco Lucarini presented a comprehensive review on the advances of research on the interdisciplinary frontier of organic radical chemistry and supramolecular chemistry.^6^ The purpose of such a fundamental research field is to combine traditional supramolecular chemistry and radical chemistry together, and take the benefit of both to eventually create new molecules and materials. On the one hand, the concept of supramolecular chemistry is expected to control and fine-tune the reactivity of organic free radicals through the various noncovalent supramolecular interactions. In fact, encapsulation of reactive species, especially organic radical cations or anions, has proven to be extraordinarily effective in enhancing their stabilities.^7^ On the other hand, organic radicals together with their distinct noncovalent spin–spin interactions can offer dramatic benefits to the diversity of supramolecular chemistry, and infuse new energy into the field of supramolecular self-assembly and advanced supramolecular materials. For example, the most important pioneering work in this field is the study of the various organic radical cation dimerizations and their host–guest chemistry and radical-based self-assembly and molecular machines.^8^ Meanwhile, organic radicals usually produce a characteristic electron paramagnetic resonance (EPR) signature which has been a powerful tool, not just for identifying the structures and properties of the noncovalent assemblies, but also for unveiling their self-assembly mechanism.^9^

**Fig. 1:**
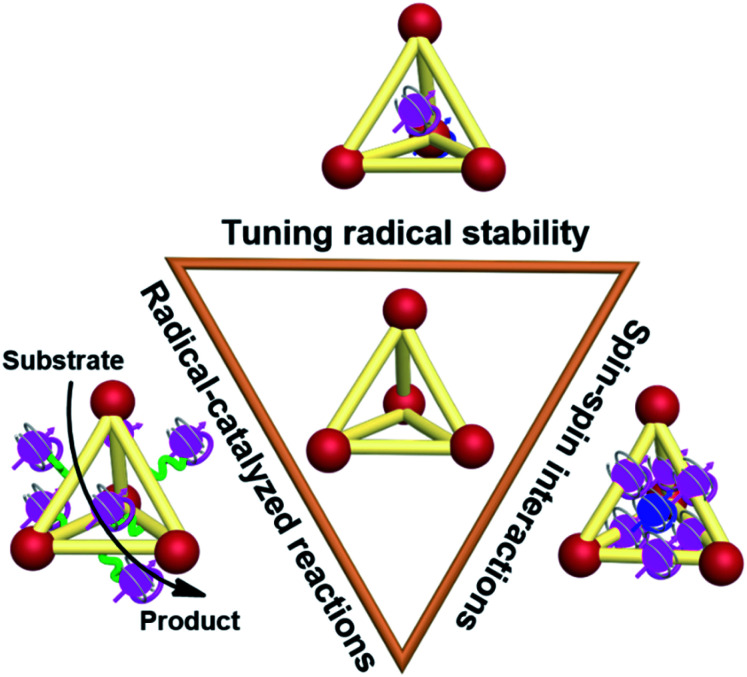
Cartoon representation of the main topics of supramolecular radical cages, modulation of radical stabilities, spin–spin interactions and application in radical-mediated/catalyzed reactions, discussed in this *Perspective*.

Recently, the incorporation of organic radical units into supramolecular cages, namely supramolecular radical cages, has given new vitality to supramolecular chemistry since the radicals within a specific three-dimensional (3D) cage will lead to some interesting properties and applications to this specific supramolecular radical system. The unique 3D topological structures and the confined nanospaces of supramolecular cages are expected to have a pronounced effect on the radicals' properties such as their stabilities, spin–spin interactions and the related radical-mediated/catalyzed reactions (Fig. 1). For example, some novel covalent or coordinated supramolecular cages consisting of radical centres or redox-active building blocks have been successfully developed in spite of their molecular design and synthesis being extremely challenging. Notably, the radical species can be arranged in an orderly manner in the well-defined cage structures, which is conducive to inducing the intriguing spin–spin interactions between radical species through space or through bond within a specific distance. Besides, encapsulation of paramagnetic guest molecules (*e.g.*, organic radicals and paramagnetic metal ions) within the interior cavities of supramolecular cages is also of great interest in this field. Some representative studies have revealed that the confined nanospaces of supramolecular cages can efficiently regulate the properties of radical guests such as their stabilities and spin–spin interactions. Moreover, chemists have also successfully employed radical cages or introduced reactive radical species into the cage cavity to catalyze some reactions that are difficult to carry out under normal conditions.

Our group has recently engaged in this field and we think that the area of supramolecular radical cages still remains largely unexplored.^10^ A large exploration research space exists in this topic. We would like to summarize and highlight the recent progress on the development of supramolecular radical cages regarding their design and synthesis, chemical and physical properties and applications. Some other prevailing topics in supramolecular radical chemistry, such as radical cationic host–guest complexes and molecular machines,^11^ applications of radical cation dimerization in self-assembly,^12^ supramolecular radical polymers,^13^ applications of EPR techniques in supramolecular chemistry,^9*a*^ supramolecular strategy for preparing stable radicals,^7*a*^ organic radical-based dynamic covalent chemistry^14^ and so on have already been summarized in other excellent reviews, and will thus be excluded in this article. The main content of this *Perspective* will contain three sections: radical cages, radicals in a cage, and cage-confined radical-mediated/catalyzed reactions. In the end, we will offer some perspectives on the challenges and outlooks in this emerging area, particularly with respect to the new molecular design and synthesis, mechanism study and application, which may be helpful for people who are or will be engaged in this field.

## 2. Radical cages

**Fig. 2:**
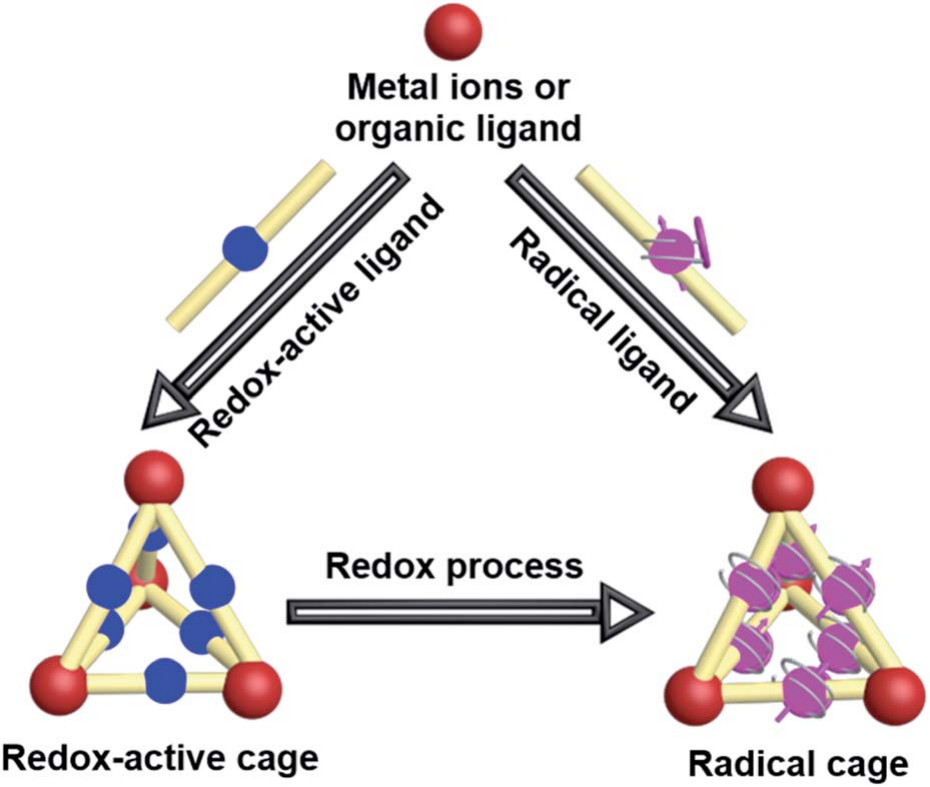
Cartoon representation of the general synthetic strategies for the design of radical cages.

The intrinsic properties of organic radicals together with the relatively complicated structures of cages make the design, synthesis, purification and characterization of radical cages extremely difficult. Nevertheless, a number of novel radical cages have been successfully developed in the past few decades. The general synthetic strategies for the design of radical cages can be divided into two kinds of method, namely “constructing a cage and then generating a radical” and “synthesizing a radical and then constructing a cage” (Fig. 2). Specifically, “constructing a cage and then generating a radical” refers to the construction of molecular cages bearing some redox-active units or some precursors of radicals firstly, which subsequently can be readily converted to the corresponding radical cages, *via* reduction/oxidation, irradiation, or direct heating. In contrast, “synthesizing a radical and then constructing a cage” includes the design and synthesis of some stable radical building blocks firstly, and then the self-assembly of radical cages based on these radical building blocks. In addition, a few π-conjugated radical (radicaloid) cages have also been successfully obtained *via* multi-step synthesis. Notably, the difficulties in the development of novel radical cages are mainly reflected in their chemical instability that requires special attention. Moreover, the precise characterization of radical cages including their structures and radical properties often relies on many advanced characterization techniques, among which the most direct one is X-ray crystallography, but for radical cages the single crystal growth is always a big challenge.

### 2.1 Redox-active cages

**Fig. 3:**
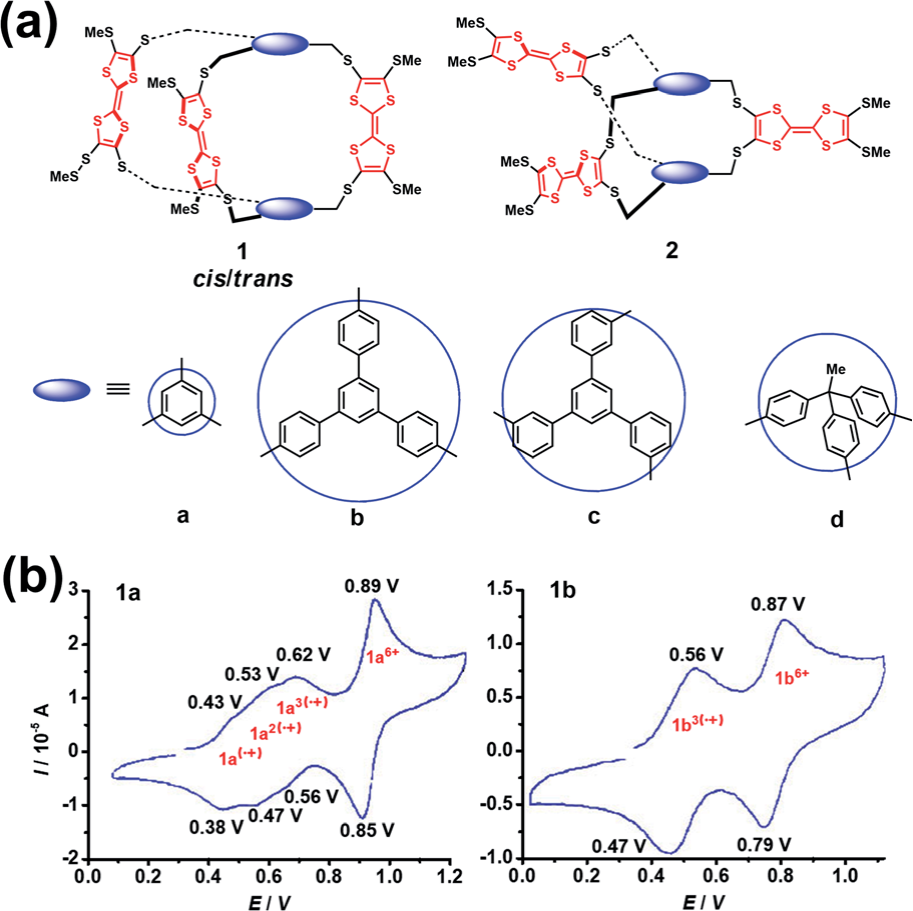
(a) Structures of a series of tetrathiafulvalene-bridged cages (**1a–1d** and **2a–2d**). Reproduced from ref. 17. (b) Cyclic voltammograms for compounds **1a** and **1b**. Reproduced from ref. 17.

The construction of cages containing redox-active units, such as ferrocenyl, viologen dication, tetrathiafulvalene (TTF) and so on, is of great interest in supramolecular chemistry since such systems exhibit very intriguing redox properties.^15^ In addition, redox-active cages in principle can electrochemically regulate the host–guest interaction with any charged or neutral guest.^16^ Notably, one of the main issues and challenges of the synthesis of cages bearing polyradical cations or anions is the non-negligible electronic communication between each of the redox-active units that makes the oxidation or reduction of redox-active units, especially the adjacent redox-active units, more difficult. Because the Coulomb repulsion is highly dependent on the distances between the redox-active units, the redox activity of cages is thus varied by changing the dimensions and rigidity of the spacer units. Generally, the separated redox-active units usually have negligible electronic communication, and thus they exhibit identical redox properties, *e.g.*, redox potential, and chemical and electrochemical reversibility. In contrast, the adjacent redox-active units have considerable Coulomb repulsion, and the repulsive Coulomb force significantly affects the original redox properties of the redox-active units. In this scenario, the redox-active units in a cage tend to undergo stepwise redox processes and show multiple separated redox peaks in the cyclic voltammograms. For example, Becher *et al.* successfully developed a series of macrobicyclic tetrathiafulvalene-bridged cage molecules (**1a–1d** and **2a–2d**) *via* an ingenious synthesis strategy involving the facile protection–deprotection of TTF-thiolates and the subsequent *in situ* alkylation (Fig. 3a).^17^ These TTF-based cages exhibited very distinctive redox properties by the analysis of their cyclic voltammetry. Taking **1a** and **1b** as examples (Fig. 3b), cage **1b** possessing a large spacer between the TTF units exhibited two well-defined three-electron reversible redox waves corresponding to the simultaneous formation of three radical cations followed by three dications at higher potentials. In contrast to the one-step three-electron redox process of **1b**, **1a** wherein the TTF groups were linked by the shorter spacer of 1,3,5-trimethylenebenzene group underwent a stepwise one-electron oxidation process involving a total of three electrons accompanying the formation of a mono-, a bis-, and a tris(radical cation) species. However at higher potentials a simultaneous loss of three electrons of the tris(radical cation) (**1a**^3(^˙^+)^) gave rise to the six-fold charged tris(dicationic) state, as commonly observed in these series TTF-based cages. The difference in the redox behaviour between **1a** and **1b** was interpreted to be closely correlated with their structures, *i.e.*, a close proximity of the three redox TTF moieties in **1a** induced significant through-space coulombic interactions, while the large and rigid aromatic spacers in **1b–d** prevented such coulombic through-space interactions among the three TTF groups. Notably, no suitable single crystals of radical cation species of these series TTF-based cages for single crystal X-ray diffraction (XRD) can be obtained, mostly because of the stability issues and strong Coulomb repulsion of the polyradical cations.

**Fig. 4:**
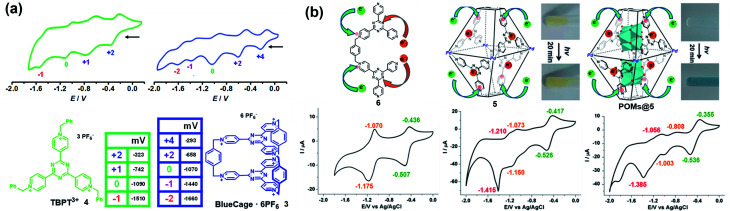
(a) BlueCage^6+^ (**3**), TBPT^3+^ (**4**) and their corresponding cyclic voltammograms. Reproduced from ref. 18. (b) Cyclic voltammograms of ligand **6**, cage **5**, and POMs@**5** with corresponding oxidation states for each reduction wave and the photochromic photographs of cage **5** and POMs@**5** before and after irradiation. Reproduced from ref. 19.

Similarly, some pyridinium-based cages and their redox behaviours have also been investigated. For example, Stoddart and co-workers reported the template-directed synthesis of Blue-Cage^6+^ (**3**), a macrobicyclic cyclophane consisting of six pyridinium units and two central triazines which are both redox-active moieties (Fig. 4a).^18^ The electron-deficient nature of **3** endowed it with interesting molecular recognition towards polyaromatic hydrocarbons (PAHs) as well as PF_6_^−^ ions. Moreover, cage **3** exhibited five reversible reduction waves, wherein the first three appeared at low electrode potentials indicating the three two-electron reduction processes accompanying the formation of bis-, tetra- and hexa-pyridinyl radicals. Interestingly, cage **3** and its half-cage analogue TBPT^3+^ (**4**) featured similar potentials for the first three reductions, thus indicating the lack of electronic communication between the two tritopic platforms of the cage during the reduction process. In contrast, the reduction of the central triazines experienced a stepwise one-electron reduction process and the reduction potentials were more negative than that of **4**, implying the non-negligible electronic communication between the two central triazines. Again, based on the pyridinium and triazine units, Sun *et al.* reported a water-soluble redox-active supramolecular Pd_4_L_2_ molecular cage **5** (Fig. 4b).^19^ Unlike the relatively low yield synthesis of cage **3**, cage **5** was efficiently constructed by coordination-driven self-assembly of four *cis*-blocked palladium corners and two pyridinium-functionalized bis-bidentate ligands (**6**). The electron-deficient **5** featuring enlarged pore-openings and internal cavities demonstrated its great potential for application in the encapsulation of aromatic molecules and polyoxometalate (POM) catalysts. Interestingly, POMs@**5** complexes showed enhanced photochromic behavior compared to that of cage **5**, mainly attributed to the charge transfer interaction between the electron-rich POM donor and electron-poor pyridinium acceptors in **5** (inserted figures in Fig. 4b). Similar to cage **3**, the cyclic voltammetry of cage **5** indicated that the formation of a cage had little effect on the reduction process of pyridinium moieties when compared with its half-cage ligand **6**, but significantly affected the redox behaviour of triazine units because of the considerable interligand electronic communications between the triazine panels. In addition, the encapsulation of POMs within cage **5** was demonstrated to have a profound effect on its redox behaviour, *e.g.*, the three redox waves of the inclusion complexes POMs@**5** were shifted to more positive potentials and became quasi-reversible or totally irreversible compared with those of cage **5** (Fig. 4b). Similar to TTF cages, it is extremely challenging to gain insight into each radical species for cages **3** and **5** directly by single-crystal X-ray diffraction, on account of the unstable nature of the pyridinyl radical and triazine radical anion. Notably, the role of the electrolyte is often ignored during the electrochemical investigations of redox-active cages in many reports. The electrolyte as a counter guest is expected to play an important role in the stabilization of radical species as well as the mediation of the through-space electronic communications between radical components and the charged host cages.^20^ However, the molecular-level understanding of the role of counter anions is very difficult due to the limited examples of X-ray single-crystal analysis of redox-active cages bearing multiple radical ions.

**Fig. 5:**
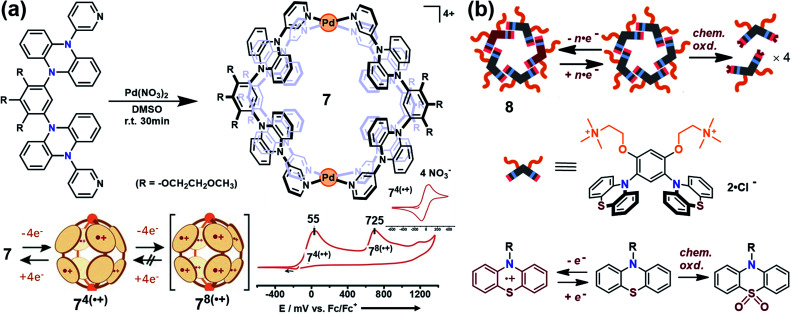
(a) Schematic representation of the formation of **7** and its proposed transformations by the sequential oxidation/reduction processes and cyclic voltammograms. Reproduced from ref. 22. (b) The redox-active supramolecular capsule **8** with multiple phenothiazine panels and the three different states of phenothiazines. Reproduced from ref. 23.

Yoshizawa and co-workers have successfully developed an ingenious and versatile strategy to construct supramolecular capsules based on bent polyaromatic building blocks either by a coordination approach or through a π-stacking approach.^21^ Conceivably, the utilization of the redox-active polyaromatic panels could bring some interesting redox properties to the resultant supramolecular capsules. For example, an M_2_L_4_ capsule (**7**) containing eight redox-active, dihydrophenazine panels was quantitatively obtained by a coordination approach (Fig. 5a).^22^ Electrochemical studies revealed that capsule **7** featured a stepwise four-electron oxidation process wherein the first oxidation wave was reversible while the second one was irreversible, indicating that the formation of tetra(radical cation) capsule **7**^4(^˙^+)^ was feasible and reversible while the octa(radical cation) capsule **7**^8(^˙^+)^ was much more reactive and difficult to acquire. Therefore, the structure of **7**^4(^˙^+)^ was reasonably interpreted as a tetra-positive spherical shell consisting of four monoradical and four neutral panels arranged alternately. The clearly separated first and second four-electron oxidations could be attributed to the considerable coulombic through-space interaction, which also made the second oxidation more difficult and the tetra(radical cation) species more reactive. Likewise, self-assembly of a redox-active supramolecular capsule **8** based on the bent phenothiazine panels through the hydrophobic effect and π-stacking interactions was also reported by the same group (Fig. 5b).^23^ Both electrochemical and chemical oxidation of capsule **8** produced relatively stable radical cation species **8**^*n*(^˙^+)^ at room temperature in a reversible fashion. Capsule **8** exhibited the host capability to encapsulate guest molecules like pigment blue 15 and fullerene C_60_ in water, and subsequent chemical oxidation of the products generated radical host–guest complexes still with reasonable stability. Meanwhile, phenothiazine moieties could be quantitatively converted to sulfoxide in the presence of oxidant NaClO, leading to the disassembly of capsule **8** into the monomeric species as well as the release of bound guests from the capsule cavity.

### 2.2 Self-assembled radical cages

Though the above-mentioned redox-active cages have presented their capability to generate radical cages, such “constructing a cage and then generating a radical” strategy encounters the main problem of the precise generation and characterization of radicals since the multiple redox states are usually very difficult to control and the resultant multiple radical species are normally unstable. Therefore, an alternative strategy, namely “synthesizing a radical and then constructing a cage”, has been widely employed to achieve radical cages (Fig. 2). Generally, such a method involves the design and synthesis of a stable radical which can serve as a ligand, and the subsequent radical cage formation, often through the self-assembly approach. Thus, the rational design of suitable organic radical ligands with well-defined geometry and persistent stability is the prerequisite to realize the construction of radical cages. Indeed, organic radical ligands not only dictate the structure and topology of the resulting radical cages but also determine their properties and applications. Moreover, the radical centers could be arranged in an orderly manner in a well-defined cage, which is conducive to studying the interaction between radical species through space or through bond within a specific distance.

**Fig. 6:**
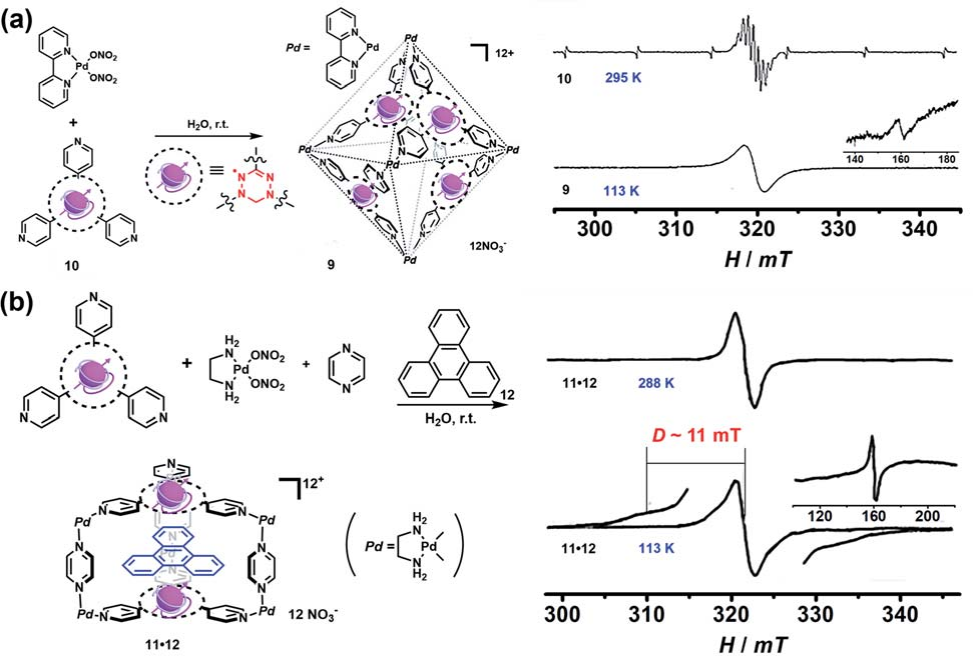
(a) Self-assembly of M_6_L_4_-type radical cage **9** and the corresponding EPR spectra of ligand **10** and cage **9**, inset shows Δ*M*s = 2. Reproduced from ref. 24. (b) Self-assembly of prism-shaped radical cage **11** and the EPR spectra of complex **11**·**12** in 295 K and 113 K, inset shows Δ*M*s = 2. Reproduced from ref. 25.

In 2008, Fujita and co-workers reported a self-assembled M_6_L_4_ radical cage **9** containing four spin centers around the cavity (Fig. 6a).^24^ Radical cage **9** was quantitatively formed *via* the self-assembly of four verdazyl radical ligands (**10**) and six palladium corners. Because the *C*_2v_-symmetric **10** in principle could generate ten possible structural isomers of cage **9**, single crystal XRD disclosed that cage **9** inevitably involved severe disorder but the cage structure was unambiguously confirmed. Notably, the four radical centres of cage **9** showed interesting intramolecular spin–spin interactions, as evidenced by its obviously broad EPR signal in contrast to the well-resolved nine sharp signals of ligand **10**. The observation of a forbidden half-field transition (Δ*M*_S_ = 2) also supported the presence of intramolecular spin–spin interactions. Subsequently, a prism-shaped radical cage **11** was also reported by the same group (Fig. 6b).^25^ Unlike cage **9**, the single crystal of cage **11** was not disordered and encapsulated one template molecule of triphenylene (**12**), wherein the two verdazyl panels were parallel and rotated by 120°. Similar to cage **9**, significant magnetic interactions were also observed between the two verdazyl panels in cage **11** as proven by the broadened EPR signal. Unlike cage **9**, zero-field splitting (ZFS) arising from direct spin–spin dipole–dipole interaction was observed in the EPR of **11**·**12** at a lower temperature, mainly because of the relatively shorter distance between the two radicals. The splitting of signals with *D* = 11 mT was well consistent with the distance between the two coupled verdazyl panels based on point dipole approximation (PDA). In addition to the intramolecular magnetic interactions, **9** and **11** also exhibited intriguing noncovalent host–guest magnetic interactions through the encapsulation of open-shell species, which will be discussed in the following *Radicals in a cage* section. Recently, a networked radical cage consisting of verdazyl radical ligands (**10**) and Co(ii) ions was also reported by Loh's group, which demonstrated very distinct magnetic interactions between the Co(ii) ion center and radical ligands as well as between the host radical cage and the guest molecules.^26^

**Fig. 7:**
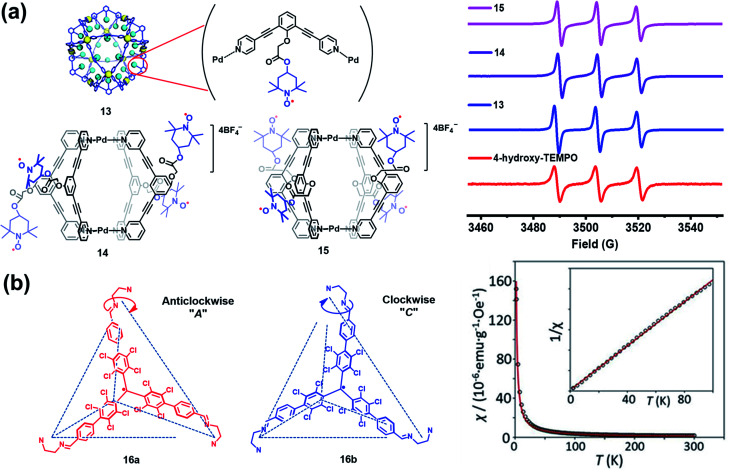
(a) The structures of TEMPO radical-functionalized cages **13**, **14** and **15** and their solution state EPR spectra. Reproduced from ref. 27. (b) Structures of the chiral radical cage **16** with two enantiomers of **16a** and **16b** and its SQUID data measured at an applied field of *H* = 3000 Oe. Reproduced from ref. 28.

Besides verdazyl radicals, some other stable organic radicals such as TEMPO and polychlorotriphenylmethyl (PTM) radicals have also been used as ligands for the construction of radical cages. In 2017, Fujita's group reported the self-assembly of an M_12_L_24_ radical cage (**13**), wherein twenty-four TEMPO spins were encapsulated within the cavity of cage **13** (Fig. 7a).^27^ Cage **13** together with another M_12_L_24_ cage **40** (Fig. 13a) bearing MacMillan's catalyst can catalyze a stereoselective cascade reaction (allylic oxidation followed by Diels–Alder cyclization) that is difficult to carry out under normal conditions because MacMillan's catalyst is vulnerable to the TEMPO oxidant (*vide infra*). The EPR spectrum of cage **13** in the solution state was surveyed in our group, showing a characteristic three-line signal similar to that of the free TEMPO unit (Fig. 7a). The isotropic hyperfine splitting pattern and the unchanged *A*_N_ value probably implied that the twenty-four TEMPO spins were not strictly confined within the cavity of cage **13**. Recently, we prepared a series of TEMPO radical-functionalized supramolecular coordination complexes including metallacycles and metallacages, wherein the number, location, and distance of the spins were precisely controlled.^10^ Their intriguing spin–spin interactions were systematically investigated by EPR and were well interpreted at the molecular level assisted by X-ray crystallography analysis. Particularly, the *exo*- and *endo*-TEMPO radical-functionalized cages (**14** and **15**) exhibited some distinctive properties. For example, the proton signal in the NMR spectrum of cage **15** became much broader compared to that of cage **14**, probably because of the more concentrated paramagnetic environment of the *endo*-TEMPO radical-functionalized cage. However, **14** and **15** featured a similar EPR profile of a three-line pattern due to *m*_I_ = 0, ±1 and *A*_N_ ≈ 15.7 G, also similar to that of **13**. X-ray crystallographic analysis disclosed that cage **15** featured a lantern-shaped conformation, wherein the four *endo*-TEMPO spins were stretched entirely outside of its cavity (Fig. 7a), which supported its unchanged *A*_N_ value and unexpected weak spin–spin interactions. This finding may offer some support for our speculation that TEMPO units in cage **13** were not strictly restricted and might also partially stretch out of the cavity. Very recently, Jiao, Cao, Li and co-workers reported a purely covalent radical cage (**16**) containing four PTM spins *via* dynamic covalent chemistry (DCC).^28^ An interesting chiral self-sorting behaviour in the cage formation was observed, and the two enantiomers **16a** and **16b** were successfully separated by chiral high-performance liquid chromatography (HPLC) (Fig. 7b). Similar to cage **9**, intramolecular magnetic interactions between four PTM spins were also observed in cage **16**. Superconducting quantum interference device (SQUID) measurement for cage **16** further indicated that the four spins underwent weak coupling within the cage and almost exhibited independent paramagnetic behaviour (Fig. 7b), primarily because the two adjacent PTM radicals had a relatively large distance (9.74 Å).

### 2.3 π-Conjugated radical (radicaloid) cages

Most of the aforementioned self-assembled radical cages are based on the well-developed stable radicals, and the spin density thereof is mainly localized on the radical units rather than the whole cage framework. Moreover, the radical ligands are generally linked by some diamagnetic metal ions or non-conjugated spacers, which is very unfavorable for the intramolecular spin–spin communication. Therefore, the through-space spin–spin interactions often dominate the magnetic interaction within the framework of radical cages, which are strongly distance- and conformation-dependent. In this scenario, the synthesis of π-conjugated radical cages is of great interest since some distinct magnetic interactions are expected to be appealing in this conjugated system.

**Fig. 8:**
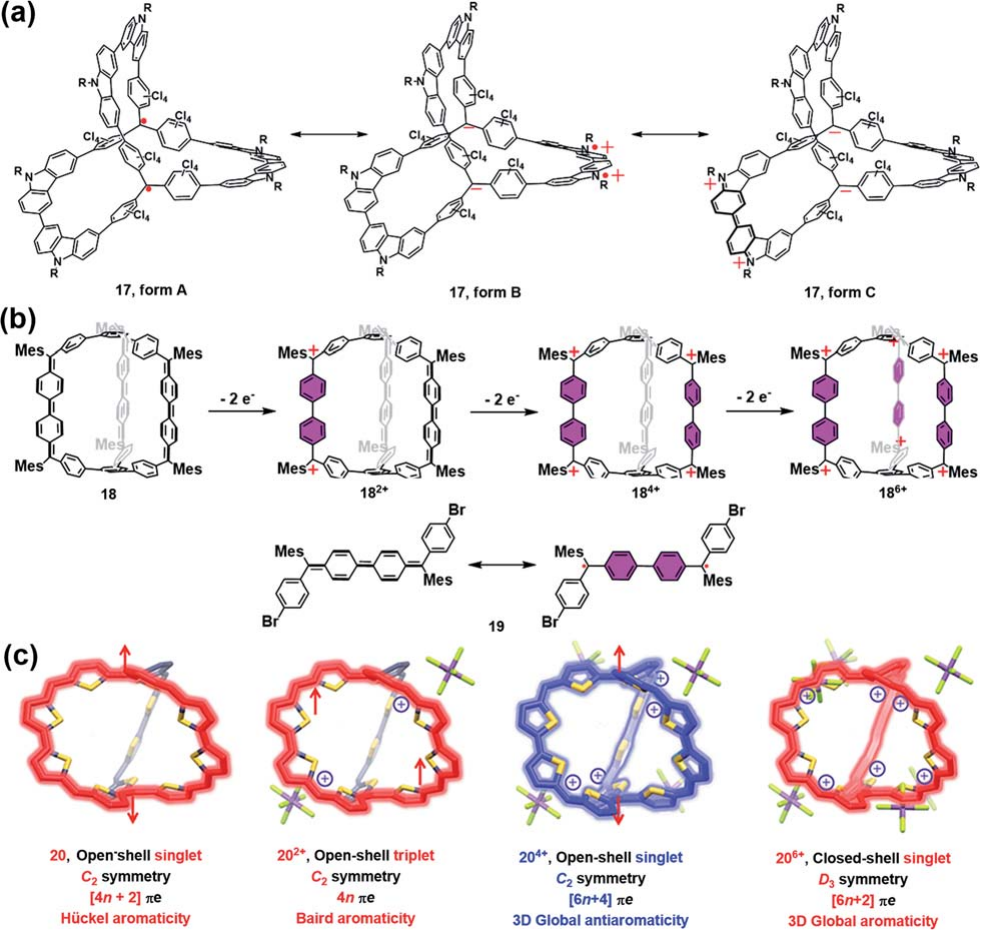
(a) Three dimensionally π-conjugated diradical molecular cage **17** and its three typical resonance forms. Reproduced from ref. 30. (b) Chichibabin's hydrocarbon (CH) based radicaloid cage **18** and its three oxidation states (**18**^2+^, **18**^4+^ and **18**^6+^), followed by the resonance of CH analogue **19** on the bottom. Reproduced from ref. 31. (c) Three-fold symmetrical diradicaloid cage **20**, its three oxidation states (**20**^2+^, **20**^4+^ and **20**^6+^), the corresponding π-electron delocalization pathways and the applied aromaticity rules. Reproduced from ref. 32.

Wu and co-workers have done pioneer work in this area and successfully developed several π-conjugated radical cages and radicaloid cages.^29^ In 2017, they reported a three-dimensionally π-conjugated diradical molecular cage (**17**) synthesized *via* multiple steps involving a main step of intermolecular Yamamoto homo-coupling.^30^ Theoretically, three typical resonance forms can be drawn for cage **17**, *i.e.*, a pure open-shell configuration (form **A**), an open-shell zwitterionic structure (form **B**), and a closed-shell quinoidal zwitterionic form (form **C**), depending principally on the manner of spin communication in **17** (Fig. 8a). The spin-unrestricted density functional theory (DFT) calculations indicated that the diradical character index *y*_0_ of cage **17** was approximately 0.99, suggesting very weak coupling between the two spins. SQUID measurement of microcrystals of **17** showed a *χ*_M_*T* value of about 0.6 emu kmol^−1^ at 300 K, which was lower than the theoretical value of 0.75 emu kmol^−1^ for the two uncorrelated *S* = 1/2 spins, and much smaller than the expected value for the triplet ground state biradical (∼1 emu kmol^−1^). The relatively lower magnetization of cage **17** was interpreted to be on account of the solvent residue. Bleaney–Bowers equation fitting results further implied that cage **17** could be viewed as nearly pure diradical with degeneracy of singlet and triplet states, due to the very weak spin communication. Similar to the synthetic method of **17**, a three-dimensional π-conjugated polyradicaloid molecular cage (**18**) was successfully obtained by the same group.^31^ Cage **18** consisted of three Chichibabin's hydrocarbon (CH) motifs which were connected by two benzene-1,3,5-triyl bridgeheads (Fig. 8b). Because Chichibabin's hydrocarbon represents one of the most classical open-shell radicaloids, the resultant cage is referred to as a “radicaloid cage”. Theoretic calculation results showed that three CHs in cage **18** were nearly decoupled due to the cross-conjugated 1,3,5-linkage mode of 1,3,5-triphenylbenzene units. As a consequence, multiple diradical characters with *y*_0_ = 0.67, *y*_1_ = 0.66, and *y*_2_ = 0.51 for cage **18** were determined by the natural orbital occupation number (NOON) calculations. Significantly, the *y*_0_ value for cage **18** was slightly smaller than that of CH analogue **19** (*y*_0_ = 0.73), implying that structural restriction in a 3D cage structure may lead to a higher rotation barrier and a larger singlet–triplet energy gap (Δ*E*_S–T_), which was fully verified by variable temperature NMR and EPR measurements in their work. Similar to the redox-active cages, cage **18** preferred to undergo a stepwise two-electron oxidation process, involving the formation of **18**^2+^, **18**^4+^ and **18**^6+^, while the formation of the intermediate odd states (radical cation states) was difficult to control. Such a phenomenon might be correlated with the cross-conjugated mode of cage **18** in which the individual CH group was more prone to recover two aromatic sextet rings after a two-electron oxidation process.

In order to achieve a fully conjugated radical cage, Wu's group designed and synthesized a three-fold symmetrical diradicaloid cage (**20**) *via* a similar synthesis protocol to that of **17** and **18**.^32^ In particular, the thiophene ring was chosen as it has smaller resonance energy compared with the benzene ring, which facilitates the efficient delocalization of π-electrons along the entire framework of **20**. Therefore, this type of diradicaloid cage and its charged species would be an ideal platform to investigate the spin communications, wherein the distinct aromaticity and spin state were highly anticipated in this system. For instance, the neutral cage **20** was demonstrated to be aromatic following Hückel's rule (4*n* + 2 rule) and had an open-shell singlet ground state since the *C*_2_ symmetric **20** adopted a dominant 38π monocyclic conjugation pathway (Fig. 8c). With regard to its charged species, **20**^2+^ was found to have a triplet ground state and exhibited weak Baird aromaticity (Fig. 8c). Thus, the experimental and calculated results suggested a dominant 36π monocyclic conjugation pathway in **20**^2+^. **20**^4+^ was also proven to have an open-shell singlet ground state similar to neutral **20**, but overall 52 π-electrons in **20**^4+^ were fully delocalized along the entire 3D framework, suggesting a unique and strong 3D global antiaromaticity (6*n* + 2 rule) of **20**^4+^. **20**^6+^ was found to exhibit *D*_3_ symmetry and all of the 50 π-electrons were also fully delocalized, leading to the closed-shell nature and 3D global aromaticity of **20**^6+^ (Fig. 8c). The different types of aromaticity observed in this system were believed to be highly correlated with the molecular symmetry, number of π-electrons and spin communication manner in this type of diradicaloid cage and its charged species. Therefore, this work successfully demonstrates the importance of π-conjugated radical (radicaloid) cages in the fundamental understanding of 3D global aromaticity, even spherical aromaticity.

## 3. Radicals in a cage

Supramolecular encapsulation of radical(s) in a cage is of great interest since it has important practical significance in improving the stability of the reactive radical species as well as inducing some intriguing host–guest properties. On the one hand, when the radical is encapsulated in a cage, the reactivity and stability of the bound guest radical could be well tuned due to the intrinsically isolated microenvironment within a particular cage. On the other hand, one or multiple radicals confined in a (radical) cage is conducive to the through-space spin–spin interaction, even giving rise to host–guest spin–spin interactions. In addition, encapsulating a radical unit in a cage usually causes a significant change of the EPR profile of the radical guest as well as the inherent properties of the radical cage, which may give rise to some fascinating supramolecular phenomena.

### 3.1 Encapsulation of reactive radicals

**Fig. 9:**
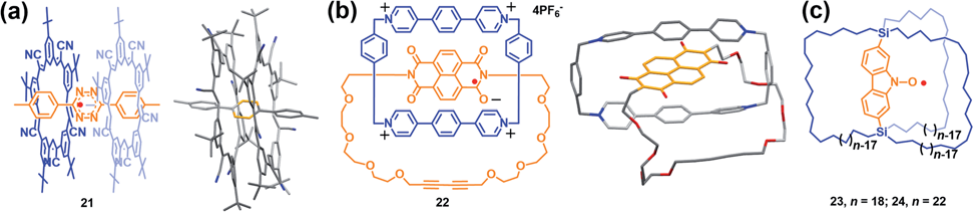
(a) Stabilizing the tetrazine radical anion using size-matched, anion-binding cyanostar macrocycles (**21**). Reproduced from ref. 36*a*. (b) Stabilizing the naphthalenediimide radical within a tetracationic cyclophane (**22**). Reproduced from ref. 36*b*. (c) Structures of the macrocages with a bridged carbazole nitroxide (**23** and **24**). Reproduced from ref. 39.

One of the main scientific issues of radical chemistry is the reactivity and stability control of organic radicals. Generally, organic radicals can be either thermodynamically stabilized or kinetically stabilized through the effective delocalization of spin density and steric protection, mainly *via* covalent approaches. Alternatively, the supramolecular strategy has also been successfully employed to modulate the stability of radicals.^7^ Actually, stabilization of reactive species or transient reaction intermediates by supramolecular encapsulation is not surprising. For instance, many studies in the field of zeolites demonstrated that short lived carbon-centered radicals became persistent when they were located inside the zeolite channels.^33^ Besides, endohedral fullerenes with nitrogen atoms and metals also highlighted the importance of encapsulation on stabilizing the reactive species.^34^ In the field of supramolecular radical chemistry, Kim *et al.* proved early that viologen and tetrathiafulvalene cation radical dimers could be stabilized within the cavity of a cucurbit[8]uril macrocycle, which shed light on the fact that supramolecular encapsulation could greatly enhance the stability of radicals.^35^ Recently, stabilization of radicals by supramolecular encapsulation in macrocycles has further proven to be a success in several reports.^36^ For example, Flood's group successfully demonstrated the supramolecular encapsulation strategy to stabilize the tetrazine radical anion using size-matched, anion-binding cyanostar macrocycles (Fig. 9a).^36^ Li, Stoddart, Wasielewski and co-workers also succeeded in stabilizing the naphthalenediimide radical within a tetracationic cyclophane (Fig. 9b).^36^ In both cases, the structures of macrocycle-radical species complexes (**21** and **22**) were unambiguously confirmed by X-ray crystallographic analysis while the individual tetrazine and naphthalenediimide radical anions were usually too reactive to achieve stable single crystal. Notably, stabilization of radicals by supramolecular encapsulation in a molecular cage remains mainly unexplored in the field of supramolecular radical chemistry. Though encapsulation of viologen and tetrathiafulvalene cation radicals within the cage was reported to enhance their stabilities, their single-crystals were not isolated.^37^ Besides, most of the documented stabilization of radicals *via* the supramolecular approach to date is based on charged radical guests. Therefore, the design of molecular cages suitable for encapsulating radical ions mainly focuses on tuning the electronic nature of cages and guest molecules, *i.e.*, very electron rich or deficient cages could facilitate the host–guest interaction with radical cations or anions, respectively, *via* electrostatic attraction and thus it is beneficial to stabilize them. In comparison, the stabilization of neutral radicals is more challenging through the supramolecular encapsulation strategy since the guest of the neutral radical is usually weakly associated with the host, making kinetic stabilization invalid.^38^ Setaka *et al.* tried to achieve kinetically neutral stabilized radicals and thus designed and synthesized macrocage molecules with a bridged carbazole nitroxide (**23** and **24**), wherein the carbazole nitroxide was covalently linked and confined in the cage (Fig. 9c).^39^ Their results indicated that the idea of kinetic stabilization of the labile carbazole nitroxide by bridging it inside a macrocage was effective. However, the radicals could not be isolated because the flexibility of the cage moieties of **23** and **24** could not protect the interior radical completely.

### 3.2 Host–guest spin–spin interactions

**Fig. 10:**
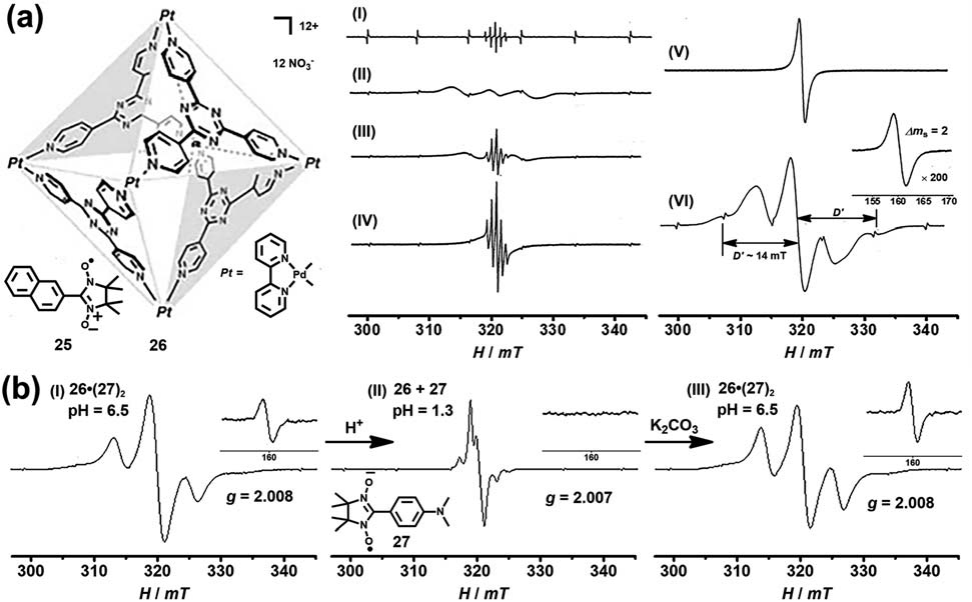
(a) The EPR spectra of host–guest complex **26**·(**25**)_2_ under different conditions ((I): **25**, solution, 293 K; (II): **26**·(**25**)_2_, frozen solution, 273 K; (III): **26**·(**25**)_2_, solution, 293 K; (IV): **26**·(**25**)_2_, solution, 363 K; (V): **25**, powder, 103 K; (VI) **26**·(**25**)_2_, frozen solution, 103 K); the inset shows the forbidden transition. Reproduced from ref. 40. (b) pH-switchable through-space spin–spin interaction of nitronyl nitroxide radical **27** within cage **26** and the EPR spectra of the host–guest complex at different pH values. Reproduced from ref. 41.

In addition to the encapsulation of reactive radicals in cages, the investigation of the unique host–guest chemistry between the (radical) cages and stable radicals is also a challenging and very significant subject. Magnetic spin–spin interactions, especially the through-space spin–spin interactions, between two or multiple radical centers are strongly distance- and conformation-dependent and, as a consequence, difficult to control. Significantly, the host–guest chemistry of radicals with cages could serve as a versatile supramolecular radical system to manipulate the through-space spin–spin interactions, which may offer exciting potential for the design of organic magnetic materials and organic spintronics. In 2004, Fujita and co-workers successfully demonstrated the manipulation of the through-space spin–spin interaction of organic radicals in the confined cavity of a self-assembled cage. Naphthylnitronylnitroxide **25**, which existed as a monomeric form in solution state, was reported to be accommodated by self-assembled cage **26** to give a stable 1 : 2 host–guest complex in solution (Fig. 10a).^40^ In this case, two **25** moieties were forced to be close to each other within the cavity of **26**. Consequently, the through-space spin–spin interaction between the two organic radicals was observed in solution as evidenced by the additional broad signal appearing in the EPR spectrum of clathrate complex **26**·(**25**)_2_ (Fig. 10a, spectrum II), in contrast to the well-resolved simple quintet EPR spectrum of guest **25** (Fig. 10a, spectrum I). The speculated intermolecular spin–spin interaction of **26**·(**25**)_2_ was further confirmed by the clear observation of a forbidden half-field transition (Δ*M*_S_ = 2) (Fig. 10a, spectrum VI), while guest **25** itself did not show that transition because the radical centers were apart from each other even in the solid state (Fig. 10a, spectrum V). The fine structure constant of *D* (∼14 mT) determined from the solid-state EPR spectrum was well consistent with the distance between the two coupled nitronyl nitroxides based on point dipole approximation. More interestingly, with the increase in temperature the fine structure constant of complex **26**·(**25**)_2_ decreased and the EPR profile became more like that of monomeric guest **25** (Fig. 10a, spectrum II–IV). This was largely because the geometry of the nitroxide radicals was fixed only by weak hydrophobic host–guest interaction and the intermolecular spin–spin interaction was very sensitive to thermal stimuli, and was thus suppressed at elevated temperature. Since the neutral nitronyl nitroxide radicals were associated with the host cage **26***via* weak hydrophobic interaction, the host–guest interaction was very sensitive to external stimuli, and the radicals easily escaped from their inclusion complex, which would significantly affect the intermolecular spin–spin interactions. On this basis, Fujita's group designed a nitronyl nitroxide radical (**27**) bearing an amine group that may be protonated and then deprotonated, and upon doing so pH-switchable through-space spin–spin interaction of organic radicals within a cage was expected to be realized (Fig. 10b).^41^ The EPR data featured a split allowed transition (Δ*M*_S_ = 1) at 321 mT and a forbidden transition (Δ*M*_S_ = 2) at 160 mT (Fig. 10b, spectrum I), suggesting the presence of the triplet species resulting from the host–guest complex **26**·(**27**)_2_. Notably, the electronic nature and hydrophilicity of **27** before and after protonation could be significantly tuned, and as a result, the release and encapsulation of radical guests from/within the cavity of the cage can be realized. As expected, when the pH was adjusted to ∼1.3 with HNO_3_, the triplet signal was completely suppressed and only a doublet signal with a hyperfine structure was observed (Fig. 10b, spectrum II), revealing the release of radical guests from the cavity of the cage. When the acidic solution was treated with K_2_CO_3_, the deprotonation process took place, regenerating the host–guest complex **26**·(**27**)_2_, so that the triplet signals reappeared (Fig. 10b, spectrum III). The release of radical guests from the cavity of the cage was mainly due to the coulombic repulsion between the positively charged cage and protonated radical species. Besides, the protonated radical guest **27** became hydrophilic and also tended to be excreted from the hydrophobic cavity of the cage. Therefore, the above two examples proved for the first time that the spin–spin interaction can be manipulated by thermal or pH stimuli reversibly, in such special supramolecular “radicals in a cage” systems.

**Fig. 11:**
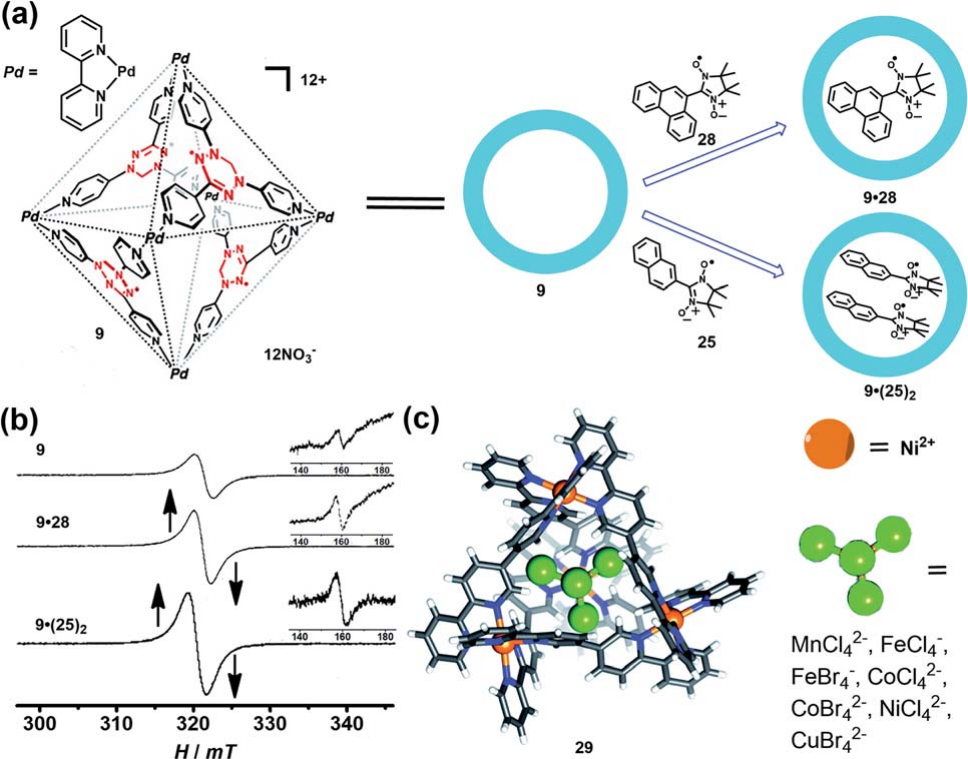
(a) Schematic representation of the formation of the radical cage-based host–guest complexes **9**·**28** and **9**·(**25**)_2_. Reproduced from ref. 24. (b) The EPR spectra of radical cage **9** and host–guest complexes (**9**·**28** and **9**·(**25**)_2_); the inset shows the forbidden transition. Reproduced from ref. 24. (c) The tetrahedral [Ni_4_^II^L_6_]^8+^ cage **29** can bind a series of paramagnetic MX_4_^1/2−^ guests. Reproduced from ref. 42.

Encapsulation of a radical guest into the cavity of a radical cage is very interesting. One can expect the intrinsic magnetic exchange interactions arising from the tunable host–guest spin–spin interactions which may produce some fascinating magnetic properties. The radical cage **9** developed by Fujita *et al.* was reported to form a host–guest complex with radical guests of **25** and **28**, resulting in clathrate complexes **9**·**28** and **9**·(**25**)_2_ (Fig. 11a).^24^ The size of guest **28** was larger than that of **25**, making cage **9** only accommodate one **28** in its cavity. Both complexes **9**·**28** and **9**·(**25**)_2_ showed a considerably enhanced Δ*M*_S_ = 1 transition as well as Δ*M*_S_ = 2 transition, compared to the empty spin cage **9**, and thus indicated the proximity of spin centers on the host and the guest in the cavity. Unlike **26**·(**25**)_2_ (Fig. 10a, spectrum VI), **9**·(**25**)_2_ showed one broad signal and a relatively weak forbidden transition signal (Fig. 11b), suggesting the presence of multiple host–guest–guest–host spin–spin interactions, in contrast to the pure intermolecular guest–guest spin–spin interactions in **26**·(**25**)_2_. SQUID measurement results revealed the antiferromagnetic properties of empty **9** and **9**·**28**, while the Weiss constant decreased from −0.1 K (**9**) to −0.4 K (**9**·**28**), implying the enhancement of antiferromagnetic spin–spin interactions by the presence of guest **28**. Such noncovalent host–guest magnetic interactions were also successfully demonstrated based on the host–guest system between the aforementioned prism-shaped radical cage **11** and open-shell metal complexes, so herein we will not repeat the details.^25^ Very recently, Brechin *et al.* presented an interesting study which indicated that the tetrahedral [Ni_4_^II^L_6_]^8+^ cage (**29**) can reversibly bind a series of paramagnetic MX_4_^1/2−^ guests such as MnCl_4_^2−^, CoCl_4_^2−^, CoBr_4_^2−^, NiCl_4_^2−^, CuBr_4_^2−^, FeCl_4_^−^, and FeBr_4_^−^, inducing distinct magnetic exchange interactions between host and guest (Fig. 11c).^42^ The magnetic exchange interactions of these series host–guest complexes were systematically investigated in their work by SQUID magnetometry, assisted by theoretical studies, disclosing that the magnetic exchange interactions between metal ions in the host complex, and between the host and guest, were of comparable magnitude and antiferromagnetic in nature. The confinement induced anisotropy of paramagnetic Co^II^ guests in this work also highlighted the potential of the supramolecular radical cage in the design of highly unusual/anisotropic single-ion magnets (SIMs).

### 3.3 Miscellaneous properties

**Fig. 12:**
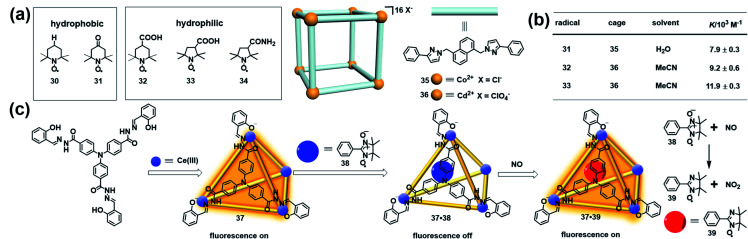
(a) The host–guest interactions between nitroxide radicals (**30–34**) and supramolecular coordination cages (**35** and **36**). Reproduced from ref. 43. (b) Calculated association constants for each radical + cage complex. Reproduced from ref. 43. (c) Structure of luminescent cage **37**, constitutive/constructional fragments of the functional cage **37** showing the sequence of its fluorescent variation upon the addition of nitronyl nitroxide radical **38** and NO, and the reaction between NO and the nitronyl nitroxide radical. Reproduced from ref. 45.

Besides stabilizing the reactive radicals and inducing distinct host–guest spin–spin interactions, encapsulation of a radical guest into a cage is usually accompanied by the emergence of some interesting properties. For example, an organic radical restricted in a confined cage usually causes a significant change of the EPR signal, which can be applied to investigate the supramolecular phenomena. Nicholas and Chechik systematically investigated the host–guest interactions between nitroxide stable radicals (**30–34**) and supramolecular coordination cages (**35** and **36**) in water and acetonitrile by means of EPR spectroscopy (Fig. 12a).^43^ Interestingly, the hydrophobic TEMPO radical **30** showed negligible association with the water-soluble cage **35**, while the same hydrophobic 4-oxo-TEMPO **31** was able to be associated with **35** with a moderate association constant of 7.9 ± 0.3 × 10^3^ M^−1^, probably due to the hydrophobic interactions and H-bonding between the cage structure and the carbonyl and nitroxide groups in **31**. Carboxylic acid-functionalized nitroxides, such as **32** and **33**, bound strongly to the acetonitrile-soluble cage **36** with association constants as high as ∼10^4^ M^−1^ (Fig. 12b). In all cases, host–guest complex formation resulted in significant decreases in the molecular tumbling rate of the guests, with tumbling becoming strongly anisotropic, as indicated in their EPR spectra. Besides, the microenvironment within the cages of **35** and **36**, including the polarity and rotational diffusion, was also successfully surveyed by EPR spectroscopy in their work. Thus, this work demonstrated that EPR spectroscopy would be an ideal technique to determine the association constants for host–guest interaction and characterize the polarity and rotational diffusion parameters of the encapsulated microenvironment.

**Fig. 13:**
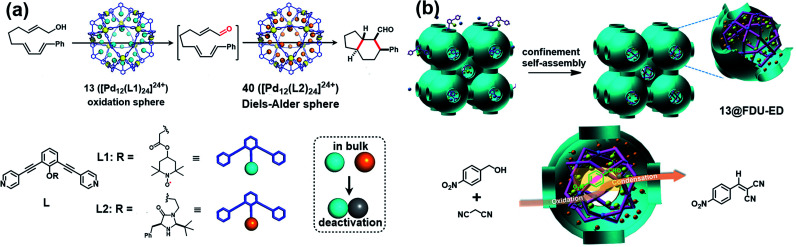
(a) The two M_12_L_24_ cages **13** and **40** bearing TEMPO and MacMillan's catalyst and their application in one-pot stereoselective cascade reaction (allylic oxidation followed by Diels–Alder cyclization). Reproduced from ref. 27. (b) Cartoon representation of the confinement self-assembly of **13**@**FDU-ED** and the application in one-pot sequential oxidation–Knoevenagel condensation reaction. Reproduced from ref. 49.

The host–guest complex of a molecular cage and organic radical in principle could function as a profluorescent radical probe if the cage is inherently emissive, similar to the working mechanism of profluorescent nitroxide probes.^44^ In 2011, Duan and co-workers developed a luminescent cage (**37**) which was capable of capturing one nitronyl nitroxide radical (**38**) to form a 1 : 1 host–guest inclusion complex **37**·**38** (Fig. 12c).^45^ Since the nitroxide radical is a strong quencher of the fluorescence, the luminescence intensity of the cage was gradually decreased with ∼90% quenching efficiency upon the addition of **38**, indicating that the host–guest complex **37**·**38** could potentially serve as a profluorescent nitroxide probe. As expected, introducing NO into the above probe immediately restored the luminescence of **37**. The EPR signal of the radical guest changed from a five-line pattern (1 : 2 : 3 : 2 : 1) to a seven-line pattern (1 : 1 : 2 : 1 : 2 : 1 : 1), indicating that the nitronyl nitroxide radical **38** reacted with NO to form imino nitroxide **39** (Fig. 12c). The fluorescence turn-on mechanism in the presence of NO was not discussed in detail in this work. One may also speculate, based on these results, that imino nitroxide **39** may be hardly associated with cage **37** and may escape from the cavity. Interestingly, this radical in a cage-based profluorescent system showed hydrophilic/lipophilic characteristics and exhibited high selectivity toward NO over other reactive species due to the special confined environment provided by the cavities of the cage, ensuring the successful application of biological imaging in living cells.

## 4. Cage-confined radical-mediated/catalyzed reactions

Supramolecular cages as artificial supramolecular catalytic systems have been extensively used for catalysis due to their relatively rigid and hydrophobic cavities that may mimic binding pockets in enzymes.^46^ Supramolecular cages, functioning as nanoreactors, have several distinct merits: (1) the confined nanospaces of supramolecular cages usually raise the local concentration of the substrate and catalyst, thus significantly accelerating the rate of reaction; (2) the reactive and labile groups of the binding motifs can be preorganized in a well-defined position, thus increasing the reaction rate and the selectivity of reactions; (3) supramolecular cages have a positive effect on stabilizing the transition state of the reaction and reducing activation energy. With the deepening of research in this field, various supramolecular cages have been successfully employed in different types of catalytic reaction, including photocatalysis, electrocatalysis, asymmetric catalysis, cascade reactions, *etc.*^47^ Organic radicals are known as important catalysts or key intermediates for catalytic reactions. Therefore, according to the above advantages, catalytic systems based on supramolecular radical cages have been developed for the related radical-mediated/catalyzed reactions. Such cage-confined radical-mediated/catalyzed reaction systems are expected to accelerate the chemical reactions or improve the reaction selectivity that is difficult to achieve under normal reaction conditions.^48^

### 4.1 Radical-catalyzed reactions

In 2017, Fujita and co-workers designed two M_12_L_24_ cages **13** and **40** bearing TEMPO and MacMillan's catalyst, respectively, and successfully solved a synthesis dilemma of one-pot stereoselective cascade reaction (allylic oxidation followed by Diels–Alder cyclization) catalysed by two intrinsically incompatible catalysts (Fig. 13a).^27^ MacMillan's catalyst is known to be promptly oxidized by TEMPO, making each incompatible with the other in such stereoselective cascade reaction. Interestingly, by encapsulating the two incompatible catalysts separately within the cavity of an M_12_L_24_-type molecular capsule, the oxidation and asymmetric Diels–Alder cascade reaction proceeded smoothly. Several control experiments indicated that only the combined use of both of the caged catalysts (**13** and **40**) allowed the desired cascade reaction to proceed, further verifying the specificity of this catalytic site isolation strategy. Recently, our group successfully developed a new bifunctional heterogeneous catalyst *via* a confinement self-assembly strategy, wherein the TEMPO-functionalized cage **13** was assembled and confined within the cavity of amino-functionalized mesoporous carbon **FDU-ED** (Fig. 13b).^49^ The unique advantages of mesoporous structures containing metal–organic structures were mainly featured in size control, site adjustment and unique confinement effects on a molecular scale, thus mimicking enzyme structures for improved organic catalysis. Consequently, the resultant heterogeneous catalyst **13**@**FDU-ED** was demonstrated to exhibit excellent stability, activity, and recyclability for one-pot sequential oxidation–Knoevenagel condensation reaction. Therefore, these two studies ideally demonstrate the versatility of the cage-confined radical-catalyst system in the continuous chemical transformations.

### 4.2 Radical-mediated reactions

**Fig. 14:**
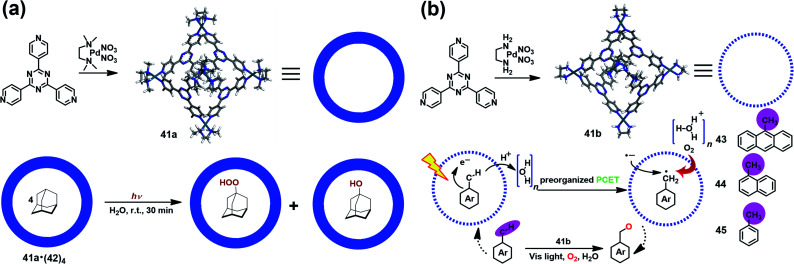
(a) Schematic representation of the radical-mediated photooxidation of adamantane **42** within a coordination cage **41a** containing a redox-active triazine core. Reproduced from ref. 50. (b) Schematic representation of the photoactivation of C–H bonds inside water-soluble nanocage **41b** and its mechanism. Reproduced from ref. 51.

Supramolecular cages consisting of redox-active moieties can readily generate radical species *via* chemical reduction/oxidation or irradiation, making them excellent in radical-mediated reactions as nanoreactors. In 2004, Fujita *et al.* successfully demonstrated the radical-mediated photooxidation of an alkane within an M_6_L_4_-type coordination cage (**41a**) containing a redox-active triazine core.^50^ Interestingly, when adamantane-encapsulated complex **41a**·(**42**)_4_ was irradiated with a high-pressure mercury lamp under aerobic conditions, guest **42** was partially converted to 1-adamantylhydroperoxide and 1-adamantanol (Fig. 14a), as indicated by *in situ* NMR. Such oxidation reaction was proven to go through a photoinduced electron transfer mechanism involving the formation of a pair of 1-adamantyl radical (plus H^+^) and radial anion of **41a**. Inspired by this work, Dasgupta and co-workers recently developed a new water-soluble photocatalytic system based on **41a** that can simultaneously preorganize the guest and polarize the C–H bonds of the guest to engineer selective functionalization (Fig. 14b).^51^ Upon illumination, a series of electron-rich alkyl-aromatic hydrocarbons, such as 9-methylanthracene (**43**), 1-methylnaphthalene (**44**), and toluene (**45**), encapsulated in cage **41b** were found to be readily converted to the corresponding neutral benzyl radicals *via* a water-assisted proton-coupled electron transfer (PCET) process, and thus activated their C–H bonds. Subsequently, the photogenerated long-lived benzyl radical within **41b** reacted with O_2_ to give the oxidized product. Their detailed mechanism studies implied that the unique and strong electric fields inside the cavity played a critical role in driving such photo-induced C–H bond activation reactions.

**Fig. 15:**
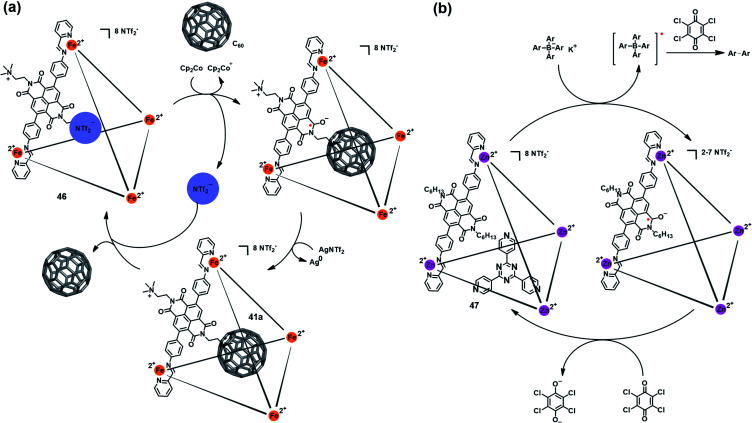
(a) Schematic representation of the reversible redox behavior of **46** accompanying a switchable anion ejection and C_60_ binding process. Reproduced from ref. 52. (b) The redox-switchable NDI-based cage **47** utilized as a catalyst for the oxidative coupling reaction. Reproduced from ref. 53.

Besides the triazine core-based self-assembled cage, naphthalenediimide (NDI)-based M_4_^II^L_6_ cages were also found to be redox-active and able to mediate a specific chemical transformation. Nitschke *et al.* reported a redox-active coordination cage Fe_4_^II^L_6_ (**46**) based on an NDI unit (Fig. 15a), which could be reversibly reduced to the radical anion state by Cp_2_Co and oxidized back to the original cage **46** by AgNTf_2_.^52^ Interestingly, such reversible redox process was accompanied by a switchable anion ejection and C_60_ binding through electron affinity reversal. A similar redox-switchable NDI-based Zn_4_^II^L_6_ cage (**47**) was developed by the same group, and successfully utilized as a catalyst for the oxidative coupling reaction of tetraaryl borates (Fig. 15b).^53^ Interestingly, the efficiency of such cage-mediated oxidative coupling reaction was highly dependent on the presence of C_60_, *i.e.*, C_60_ might serve as a radical-stabilizing agent during the catalytic process, and thus promoted the catalytic efficiency.

## 5. Conclusions and outlook

During the past few decades, a few stable and fully characterized supramolecular radical cages have been successfully developed. It is worth noting that the unique 3D topological structures and the confined nanospaces of a supramolecular cage make it an ideal platform for arranging organic radicals in an orderly manner, *i.e.*, the number, location, and distance of the organic radicals could be precisely controlled within the cage. Consequently, the self-assembled radical cages exhibited interesting and manipulatable (host–guest) spin–spin interactions, while the conjugated covalent radical (radicaloid) cages showed very unique aromaticity and tuneable electronic and spin coupling depending on their conjugation manner. Moreover, the confined nanospace of the supramolecular cage has a pronounced effect on the radical properties such as their stabilities and reactivity. For example, encapsulation of organic radicals within the interior cavities of supramolecular cages could improve the stability of the radical species. Besides, some radical mediated/catalyzed reactions have also been successfully demonstrated within coordination cages, illustrating the advantage of the supramolecular radical cage system in radical-related reactions.

Though researchers have achieved great success in the area of supramolecular radical chemistry, research on the chemistry of supramolecular radical cages is still in its infancy. On the one hand, the design and synthesis of supramolecular cages bearing stable (poly)radicals still remain a great challenge to synthetic chemists. On the other hand, the characterization of supramolecular radical cages, particularly figuring out their exact structures and intriguing (host–guest) spin–spin interactions, heavily relies on advanced characterization techniques such as single-crystal (synchrotron) X-ray diffraction, variable temperature dependent electron paramagnetic resonance (VT-EPR) and so on. Moreover, cage-confined radical-mediated/catalyzed reactions are very fancy but the choice of suitable kind of radical catalyst and reactions within a specific confined cage is very tricky and usually requires carefully molecular design and high-throughput reaction screening.

The overall research in supramolecular radical cages is still in its early stage and no one knows what advances it may bring. In our opinion, some important aspects should be considered in the future development of supramolecular radical cages. Firstly, with the aim to diversify the system of supramolecular radical cages and gain further insight into their structure–property–application relationships, a more efficient and powerful synthetic strategy is highly anticipated, which is the prerequisite to obtain various covalent (conjugated) or self-assembled radical cages. Secondly, more advanced EPR techniques such as pulsed electron–electron double resonance and electron nuclear double resonance (ENDOR) are highly expected to be used for disclosing the self-assembly mechanism of supramolecular cages and their host–guest interaction. According to our latest report and the related literature, EPR may be a very powerful tool to investigate the process and mechanism of supramolecular assembly. Thirdly, stabilizing reactive radical species through encapsulation within a supramolecular cage is an important and meaningful topic, and more efforts should be made to conduct this study. Last but not least, the development of supramolecular cages consisting of some organic dyes may have potential application in organic photoredox catalysis in organic transformations. The confined nanospaces of supramolecular cages are expected to efficiently regulate the reactivity of organic photoredox catalysis, and thus may facilitate the chemical transformations proceeding with high stereo- and regio-selectivity.

In sum, we hope that this *Perspective* will help students and researchers understand the development of supramolecular radical cages, and potentially stimulate innovation and creativity and infuse new energy into the fields of traditional supramolecular chemistry and radical chemistry as well as supramolecular radical chemistry.

## Author contributions

X. Shi and H.-B. Yang conceived the topic and structure of this perspective. B. Huang and L. Mao conducted the literature research. X. Shi and B. Huang drafted the manuscript and designed the figures. X. Shi and H.-B. Yang reviewed the manuscript.

## Conflicts of interest

There are no conflicts to declare.

## Supplementary Material
